# The influence of social value and self-congruity on interpersonal connections in virtual social networks by Gen-Y tourists

**DOI:** 10.1371/journal.pone.0217758

**Published:** 2019-06-11

**Authors:** Gonzalo Luna-Cortés, Luis Miguel López-Bonilla, Jesús Manuel López-Bonilla

**Affiliations:** 1 Fundación Universitaria Konrad Lorenz, Bogotá, Colombia; 2 Departamento de Administración de Empresas y Marketing, Universidad de Sevilla, Sevilla, España; West Pomeranian University of Technology, POLAND

## Abstract

This research focuses on the relationship of self-congruity and perceived social value with the interpersonal connections established by Generation Y tourists in virtual social networks. A quantitative study was performed using a sample of young travelers from Spain. The methodologies of Confirmatory Factor Analysis (CFA) and Structural Equation Models (SEM) were used to analyze the results. The findings of the research show that self-congruity influences the perceived social value; the perceived social value leads to satisfaction and the creation of interpersonal connections in virtual social networks; and the interpersonal connections in virtual social networks influence the use of these tools by Generation Y travelers.

## 1. Introduction

Generation Y (Millennials) is an important group of travelers in current tourism. They are young consumers who seem to feel the need for traveling and living new experiences more than previous generations did. Currently, they are a key target for many companies. Their consumer habits and the use of the new technologies might have an impact on the future of the tourism industry [1].

This group of consumers is defined as the individuals born between the beginning of the 80s and the end of the 90s [2]. Among other consumers’ motivations, it is observed in the literature that Generation Y tourists interact on their virtual social networks to create content about their tourism experiences [3]. Virtual social networks are communities of individuals who interact on specific social websites on the internet (e. g., Facebook, Instagram, Twitter, etc.), crossing geographical and political boundaries in order to pursue mutual interests [3–5].

Many Generation Y tourists create content on their virtual social networks based on the social value they perceive during their trips, and when the experience is congruent with their identities [4–8]. Thus, two constructs seem to affect the use of virtual social networks in current tourism: self-congruity and perceived social value. Self-congruity refers to the acquisition of products or brands that are consistent, enhance or, in some way, fit well with the conception the consumers have of themselves [9]; perceived social value refers to the emotional benefits acquired by the consumers through the interaction with other individuals [10].

The conclusions presented in previous studies suggest that there is a need for research regarding the motivations of Generation Y consumers when they travel, as well as the impact of virtual social networks in their decisions before, during and after their trips [3–8]. In addition, little is known about the varying types of brand–consumer interactions taking place in virtual social networks and their impact on customer value. Thus, this study can add knowledge to the current literature on perceived social value and self-congruity, by analyzing the influence of these constructs on the creation of interpersonal connections in virtual social networks among Generation Y tourists.

## 2. Literature review

### 2.1. Generation Y travelers

A generation is defined as an identifiable group that shares birth years, age, location and significant life events at critical developmental stages [1]. Regarding the relationship between consumer behavior and generational cohorts, some authors focus on the characteristics that describe each generation [2].

When it comes to the classification of each generation, most of the authors focus on the age of their members. However, there are some differences in the classification of the generations depending on the country [2].

For instance, in the studies in the USA, Generation Y is considered the group of individuals born between the end of the 70s and the middle of the 90s; while in Europe most of the authors have considered Generation Y as the group of individuals born between 1980 and 2000. In Spain, this generation is considered the Spaniards born between the years 1984 and 2000 [1–2]

In this context, Generation Y members want to have fun during their daily routine; they do not focus on reaching success as much as previous generations did; although they present themselves as an open-minded generation, whose members avoid prejudice, they show a desire to express their point of view on current subjects, regarding their lives and the behavior of others; and the opinions of their friends highly influence many of their decisions [11].

In addition, one of the most important characteristics of this generation is its connection with the new technologies. It is indicated in the literature that the internet has an impact on their consumer behavior. Members of Generation Y use virtual social networks for several reasons: sharing the experience with other members, getting information from friends and relatives, making contact with other people interested in some of their hobbies; talking about an event and trying to convince friends and relatives to attempt it; as well as other personal benefits, as, for example, having fun using their social networks [12].

Particularly, the information in virtual social networks can influence some important decisions regarding their trips [12]. This type of tourists uses their virtual social networks in order to show the products and services they acquired during their vacations. Also, they share information related to their experiences in their virtual social networks, which may reinforce their identities and make a good impression among other users [1]. These consumer behaviors are connected in the literature with two marketing constructs: self-congruity and perceived social value [4]. The following sections of the literature review focus on these topics of research.

### 2.2. Self-congruity

In 1971, Ross [9] indicated that self-congruity in consumer behavior refers to the acquisition of products or brands that are consistent, enhance or, in some way, fit well with the conception the individuals have of themselves.

In 1982, Sirgy [13] defined it as the matching of the image of the product with the consumer’s self-concept. In this research, Sirgy specified that self-congruity is a construct formed by four dimensions: *the actual self*, which indicates how the individuals perceive their *self* in reality; *the ideal self*, referring to the *self* the individuals wish to become; *the social self*, in connection with how the individuals think that others perceive their *self*; and, *the ideal social self*, associated with how the individuals desire to be perceived by others [14].

Sirgy et al. [14] created a Likert scale to measure the dimensions of self-congruity in the area of consumer psychology: actual self (e. g., I completely identify with this product/brand); ideal self (e. g., This product/brand is consistent with how I would like to see myself); social self (e. g., I identify with the people who choose this product/brand); and ideal social self (e. g., The image of this product/brand is consistent with how I would like to be seen by others). Although different methodologies have been used to measure self-congruity in the literature (e. g., personality traits [15; 16]), many studies in consumer behavior focus on the scale by Sirgy et al. [14] to measure this construct [17].

When it comes to self-congruity in the area of tourism, research indicates that this construct influences pre-trip and post-trip consumers’ intentions [18]. One of the most important consequences associated with self-congruity in tourism is the destination choice [19]. More precisely, it has been established that the higher the congruence between the destination and the individual’s self-image, the higher the intention to travel to the destination [20].

Regarding post-trip perceptions, decisions and intentions, the following constructs can be found in the literature as consequents: satisfaction [21], intention to recommend [22]; revisit intention [19]; and loyalty [18]. Furthermore, the literature provides evidence to support the relationship between self-congruity, perceived social value and other important post-trip consequences. The following section focuses on the connection of these constructs.

### 2.3. Perceived social value

The definition of value is ambiguous, as are the names expressing this concept [23]. In the business arena, value is defined as the benefits beyond those captured by their creator [24], while in consumer behavior, perceived value is conceptualized as the consumer’s overall assessment of the utility of a product (or service) based on the perception of what is given and what is received in exchange [25]. Thus, in the context of marketing, value it is perceived by the consumers, and cannot be determined objectively by the seller. Only the customer can perceive whether or not a product or a service offers value [26].

During the 80s and 90s, the most common way of measuring perceived value was the ratio or trade-off between quality and price. But, more recent authors suggest that viewing value as a trade-off between only quality and price is too simplistic [27]. Hence, different dimensions of the perceived value were presented in the field of consumer behavior. Three main dimensions are indicated in the literature: functional, emotional and social [28].

The social dimension of perceived value implies social interactions, and it especially affects consumers’ decisions to purchase leisure-based products and services [28]. According to Sweeney and Soutar [28], this construct can be measured in the area of consumer behavior focusing on three aspects: social acceptance (e. g., This product/brand helps me to feel acceptable); the importance for the consumers on how they are perceived by peers (e. g., This product/brand improves the way I am perceived by other people); and the impression made when purchasing or using the product (e. g., This product/brand makes a good impression among other people).

Sánchez et al. [29] analyzed the perceived value in the area of tourism. They included the social dimension of value and indicated that this part of the perceived value is based on the impression made among friends and relatives. Thus, through the social dimension of perceived value, consumers evaluate if the decisions made during their holidays are valued positively by the people they know.

Some recent research mentioned that there is a relationship between self-congruity and perceived social value [30]. Although this relationship was not measured directly in the study by Hosany and Martin [30], using the methodology of SEM, on a sample of 169 cruise passengers, the authors showed that self-congruity can influence the intention to recommend. Furthermore, they indicated that this relationship can be related to the association between self-congruity and social worth.

Also, Kwak and Kang [31] examined the relationship between self-congruity and perceived quality in the area of sports consumption. The authors include aspects related to the perceived social value when analyzing the perceived quality. Using a sample of 260 basketball professionals, and the methodology of SEM, the authors proved the relationship between these two constructs, which suggested an association between self-congruity and social value.

In 2013, Zhang and Kim [32] proposed a model including the constructs identity, social comparison, attitude and purchase intention. Through a regression analysis, the authors conclude that attitude and social comparison are constructs that can be connected, and both can influence attitudinal and behavioral intentions.

Based on these studies, it can be proposed that there is a relationship between self-congruity and perceived social value. It is suggested that, when the consumer’s identity and the tourism experience are congruent, the consumer will perceive a higher social value. Therefore:

H1: Self-congruity influences the social value perceived by Generation Y tourists.

When it comes to the relationship between perceived value and satisfaction, there are some studies in the literature that directly connect these two constructs. Most of the investigations include the social dimension when measuring the perceived value. Research indicates that the higher the perceived value, the higher the satisfaction [32]. Zhan and Kim [32], using the methodology mentioned above, and based on social comparison as part of the consumer’s perceived value, indicated that this construct can influence the consumer’s satisfaction. Which means, a good impression made on other people has a positive effect on the tourist’s satisfaction. Therefore, the relationship between these constructs is proposed:

H2: Perceived social value influences the satisfaction felt by Generation Y tourists.

### 2.4. Interpersonal connections on virtual social networks

According to Riper et al. [7], consumers create links on the internet to be connected after a tourism experience. Using their virtual social networks, the users share their feelings and ask for opinions [33–35]. It is observed that Generation Y members tend to create more connections on their virtual social networks than previous generations [36]. They normally engage in their virtual social networks with the consumers with whom they share common interests [36]. Particularly, they might create stronger connections with individuals with similar identities [37–38].

The connections established by the tourists in their virtual social networks were measured by Riper et al. [7] through a Likert scale (e. g., “*As a result of my holydays*, *I established new relationships with other tourists on my social networks”; “I kept in contact with other tourists I met during my trip using my social networks*”).

Using a 2 × 2 factorial design, on a sample of 330 consumers attending a luxury café establishment, Kim and Jang [4] indicated that, when there is an attachment with an experience, young consumers tend to use their virtual social networks to talk about the experience. This attachment can be influenced by the perceived congruity between the tourist’s self-concept, the experience and the identity of the people with whom they shared the experience.

Based on these previous studies, it is proposed that, the higher the congruity of the experience with the consumers’ self-concept, the higher the connections created by the consumers with other users in their virtual social networks. Based on these affirmations, the following hypothesis is proposed:

H3: Self-congruity influences the interpersonal connections on virtual social networks by Generation Y tourists.

Furthermore, Rihova et al. [6] stated that value is socially constructed and embedded in tourists’ social practices, since in the course of their social experiences, tourists bond, cement social relationships and enhance their social skills thus co-creating value. Based on a study on 336 conference attendees, and using SEM, Riper et al. [6] indicated that the higher the social cohesion perceived during the experience, the higher is the possibility to create interpersonal connections in virtual social networks with other attendees [7]. Based on these studies, the relationship between perceived social value and the interpersonal connections on virtual social networks is proposed:

H4: Perceived social value influences the interpersonal connections on virtual social networks by Generation Y tourists.

Using descriptive statistics and correlations between one-dimensional variables, on a sample of 3,079 users of virtual social networks, Cobasky [39] indicated that positive sentiments about an experience are more likely to be remembered. Using SEM, Leung and Bai [40] indicated that the tourists who are satisfied with an experience will probably want to share information, which motivates the creation of virtual relationships after the trip. Based on these findings, it can be established that the satisfaction during the experience can motivate the interpersonal connections among the tourists in their virtual social networks.

H5: Satisfaction influences the interpersonal connections on virtual social networks by Generation Y tourists.

Furthermore, in the studies by Gretzel et al. [41] and Ribeiro et al. [42], the authors suggested that there is a link between involvement and content creation. Hence, some authors affirm that highly connected consumers are more likely to spread information. Although these assertions were not examined through an empirical research in the mentioned studies, the following hypothesis is proposed based on their affirmations:

H6: The interpersonal connections in the virtual social networks influence the use of these tools by Generation Y tourists to create content.

Finally, these connections help the consumers with the production and the distribution of information. The information shared can assist other consumers with comments and opinions [42]. This occurs when some tourists use their virtual social networks to portray and reconstruct their trips, which, in consequence, generate new information for future decisions [43]. Through a simulation study, using content analysis, Xiang and Gretzel [43] affirmed that the connections among tourists in their virtual social networks positively influence its use as a source of information. Therefore, the final hypothesis is proposed:

H7: The interpersonal connections in the virtual social networks influence the use of these tools by Generation Y tourists as a source of information.

Based on the literature review, seven hypotheses are proposed in this study. Subsequently, a theoretical model is presented. The theoretical model suggests that self-congruity in tourism influences the tourists’ perceived social value, and perceived social value influences satisfaction. These three constructs affect the interpersonal connections among tourists in their virtual social networks.

**Fig 1:**
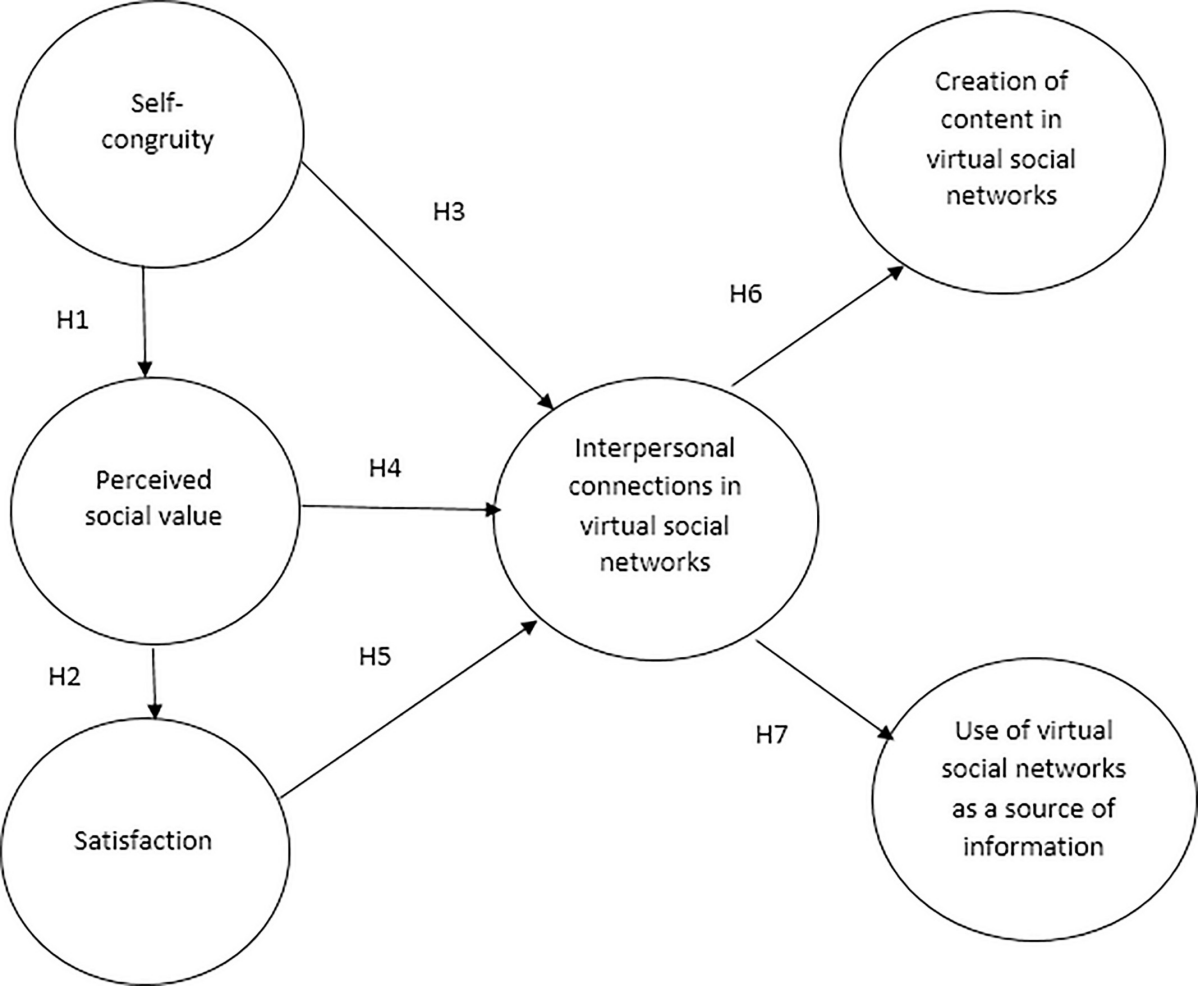
Empirical model. Source: own elaboration.

This means, the tourists might make more interactions with other users in their virtual social networks when the travel experience is congruent with their identities, and when they perceive high social value and satisfaction. Finally, the interactions among tourists influence the use of their virtual social networks to create content about their vacations and as a source of information for future decisions. The associations of these constructs are presented in Fig 1:

## 3. Methodology

### 3.1. Procedure

This study presents a quantitative research. A structured survey was used as data collection tool. A non-probability sampling was performed, among university students who recently returned from their vacation trips. The interviews were carried out on the campus of *Jerez de la Frontera*, at the University of Cadiz, in Spain. The lead author of the present study performed the interviews. These interviews were taken in Spanish, the native language of both the interviewer and the responders.

One thousand students were asked to answer the structured survey. They were asked to participate in the study in places around the campus, such as the library, the resting areas and the cafeteria. The response rate was inferior to 50%. As a result, 470 surveys were collected during February of 2016, one month after Christmas, when students had two weeks of holiday. Due to the lack of responses to some items of the questionnaire, 26 of this 470 could not be considered for the analysis. Hence, the final sample size that was used for data analysis was 444.

### 3.2. Measurement and sample

The questionnaire included the scales that measure the constructs of the research on a Likert scale of seven points (from 1-totally disagree, to 7-totally agree). The scales were chosen from previous studies in the literature of tourism and consumer behavior (see Table 1).

**Table 1:** Items of the scales.

Construct	Scale	Items
Self-congruity [SELFCON]	Sirgy et al. [1997]	• I completely identify myself with the tourism experience I lived during my last trip • I identify myself with the people who chose this trip • Living this kind of tourism experience is consistent with how I like to see myself • The experience I lived corresponds to how I like be seen by others
Satisfaction [SATIS]	McCollough et al., [2000]	• Overall, I am satisfied with the experience • This experience met my vacation needs very well • Normally, this kind of experience makes me feel satisfied
Perceived Social Value [PSV]	Sweeney and Soutar [2001]	• This tourism experience helps me to feel acceptable • This tourism experience improves the way I am perceived • This tourism experience makes a good impression on other people
Interpersonal connections on virtual social networks [IC]	Riper et al. [2013]	• As a result of my holydays, I established new relationships with other tourists on my social networks • I kept in contact with other tourists I met during my trip using my social networks • I have talked about different tourism related topics on my social networks with some other tourists I met during my trip • I value the relationships I established on my social networks with the other tourists I met during my trip
Intensity of the use of virtual social network as a source of information [ISNBI]	Ellison, Steinfield and Lampe [2007]	• My virtual social networks are main sources of information that I use in order to plan my trip • I frequently use my virtual social network to decide about my vacations • My virtual social networks have become an important source of information for the decisions I make before I travel
Intensity of the use of virtual social network to create new content [ISNCC]	Ellison, Steinfield and Lampe [2007]	• Uploading information about the tourism experience in my social network was an important part of my daily routine during and after my trip • I was proud of people reading and watching the information I uploaded in my social network about the tourism experience I lived • During my vacations and after it, using my social network to talk about the trip was an important activity for me, every day • I would be sorry if my social network shut down and I couldn´t give information about my trips

Source: own elaboration

Regarding the characteristics of the sample, all respondents were Generation Y students; 90% of them was between 28 and 25 years old. The majority of them (approximately 90%) was studying an under degree. 44% were men, and 66% percent were women.

### 3.3. Consent and translation process

Participation in the study was voluntary. The verbal consent was given individually previously to initiate each interview. The verbal consent was the first question of the interview and, if it was not obtained, the questionnaire is finished and it was not included in the study. All study participants were adults (>18 years old) and were informed about the anonymity and confidentiality of their responses. According to standard socio-economic studies, no ethical concerns are involved other than preserving the anonymity of participants. This study was approved by the Academic Committee of the Tourism Doctorate Program of the University of Seville (Spain). At that time, an official IRB (Institutional Review Board) committee had not been established at the University of Seville and the Fundación Universitaria Konrad Lorenz.

The scales used in the present research were translated from English into Spanish following a four step procedure:

1. Forward translation: the lead author of the research, who is familiar with the terminology of the area covered by the instrument, and who has interview skills, performed this task.
2. Expert panel: two researchers carefully reviewed the translations performed. Some discrepancies between the forward translation and the existing or comparable previous versions were provided, and some small changes were introduced according to the researchers’ suggestions.
3. Pre-test: a pilot survey was performed, using a sample of 30 students of the University of Cadiz, in *Jerez de la Frontera*, during the first week of February, 2016. During this pretest, the reactions, doubts and questions of the respondents were considered. A few respondents stated that they did not understand exactly what “*decision during the holidays*” and “*virtual social networks*” meant. Thus, an explanation was introduced to clearly indicate what these phrases meant (e. g., virtual social networks [such as Facebook, Instagram, Twitter, etc.]). Also, the translation of some verbs from the actual scale into Spanish resulted to technical for them. Thus, these verbs were changed into synonyms that were easier to understand by the Spanish respondents. These small changes provided more fluency during the interviews.
4. Final version: a final version of the instrument in the target language was provided after the iterations described above.

### 3.4. Data analysis

Before testing the hypotheses, the reliability and the validity of the scales were verified by confirmatory factor analysis (CFA), using EQS 6.1. First of all, Chi-Squared test, RMSEA, SRMR and GFI were examined. These are known as the Absolute Fit Indexes. They determine how well the model fits the sample data. Regarding the Chi-Squared test, a good model fit would provide an insignificant result at a 0.05 threshold.

The Incremental Fit Indexes were also examined. These are a group of indexes that do not use the Chi-Squared in its raw form but compare the Chi-Squared value to a baseline model. Research normally includes NFI and CFI [44–46].

When it comes to the analysis of the reliability and validity of the scales used in the present research, first of all, the factor loads were examined. These factors are expected to be superior to 0.60, related to significant T-values. Also, reliability was measured by Cronbach’s alpha. The threshold value of this coefficient is 0.70, what guarantees the internal consistency of the scales [47].

The composed reliability index was calculated as well in this research. The literature suggests a higher value of 0.70 to accept reliability regarding this index [48]. In addition, the average variance extracted (AVE) was calculated. Regarding this test, values over 0.50 should be obtained for every construct to accept reliability [47].

Discriminant validity was examined through two tests: firstly, the confidence interval for the inter-factor correlations was measured. In order to accept the discriminant validity, the 95% confidence intervals for correlation estimations between the pairs of factors should not contain the value “1”. Secondly, the variance shared by each pair of factors was calculated. To accept discriminant validity, these values need to be below their corresponding variance extracted indexes [47].

Subsequently, the methodology of the structural equation models (SEM) was used with EQS 6.1, to evaluate the structural model and to estimate the set of coefficients for the causal relations between variables. More precisely, a Covariance Structure Analysis was performed, using Maximum Likelihood as estimator (SEM-ML).

Based on the research by Bollen and Pearl [49], researchers should not derive causal relations from SEM. More precisely, the authors indicate that the assumptions need to be derived from (p. 309): “…*the research design*, *prior studies*, *scientific knowledge*, *logical arguments*, *temporal priorities*, *and other evidence that the researcher can marshal in support of them*.” The authors also explain that (p. 309) “…*The credibility of the SEM depends on the credibility of the causal assumptions in each application*”. According to the foundations by Bollen and Pearl [49], it should be indicated that the causal relations that are intended to be proven in the present research are based on the previous studies, which were discussed during the literature review, and not exclusively on the results provided by the SEM study.

## 4. Results

### 4.1. Model fit

First of all, regarding the model fit index of the model built in this study, the following indices were examined: Normed fit index (NFI); Comparative Fit Index (CFI); Incremental Fit Index (IFI); Goodness-of-fit index (GFI); Adjusted goodness-of-fit index (AGFI); Root mean square error of approximation (RMSEA). In order to accept good fit, these indices should be higher than a series of values recommended in the literature [44]: NNFI > 0.90; CFI > 0.90; GFI > 0.90; RMSA < 0.08; SRMR < 0.08.

It is important to mention that, at the beginning, the model fit indexes showed lower values than the recommended in the literature. Thus, the items of the scales were revised.

It was detected that the fourth item on the Intensity of the use of Virtual Social Networks to Create Content (INSCC4) and the third item on the Perceived Social Value Scale (PSV3) presented factor loadings lower than the rest of the items in these scales.

The Lagrange test suggested removing these items in order to improve the model fit [47]. The items were removed. This means, one item in the scale by Ellison, et al. [50] to measure *the use of virtual social networks to create content*, and one item of the scale by Sweeney and Soutar [28] to measure *the perceived social value*, were not consistent with the rest of the items to examine these constructs in the context of research of the present study (a sample formed by Generation Y travelers from Spain). After these changes, the measurement model showed a good fit (see the last row of Table 2).

**Table 2:** Reliability and convergent validity.

Factor	Indicator	Load	Robust t	Cronbach´s α	CR	AVE
Satisfaction	SATIS1	0.91**	15.683	0.759	0.71	0.67
	SATIS2	0.88**	14.526			
	SATIS3	0.64**	13.325			
Self-congruity	SELFCON1	0.73**	10.846	0.724	0.74	0.71
	SELFCON2	0.72**	8.074			
	SELFCON3	0.95**	14.772			
	SELFCON4	0.95**	16.577			
Perceived Social Value	PSV1	0.83**	24.100	0.822	0.73	0.53
	PSV2	0.84**	24.100			
Interpersonal Connections on Virtual Social Networks	IC1	0.62**	15.137	0.937	0.71	0.67
	IC2	0.87**	24.178			
	IC3	0.90**	25.232			
	IC4	0.86**	23.704			
Use of Virtual Social Networks as a source of information	VSNBI1	0.78**	16.183	0.766	0.70	0.65
	VSNBI2	0.82**	16.965			
	VSNBI3	0.82**	16.965			
Use of Virtual Social Networks to create content	VSNCC1	0.78**	16.988	0.799	0.68	0.63
	VSNCC2	0.80**	17.318			
	VSNCC3	0.80**	15.137			

N = 444

**p<0.01

*p<0.05

CHI-Square = 3.067.936; df = 989; NNFI = 0.932; GFI = 0.913; CFI = 0.908; RMSEA = 0.076; SRMR = 0.079. Meaning of acronyms in Table 2: Degrees of freedom (df); Normed fit index (NFI); Comparative Fit Index (CFI); Goodness-of-fit index (GFI); Root mean square error of approximation (RMSEA); Standardized root mean residual (SRMR). Source: own elaboration

### 4.2. Reliability and validity of the scales

In addition, the reliability and convergent validity of the scales are presented in Table 2. The table includes the factor loading, as well as the T-values, of every item (third and fourth columns in the table), the Cronbach’s alphas regarding every scale (fifth column), the composed reliability index (sixth column) and the average variance extracted (last column of the table).

After these changes, convergent validity was confirmed. All the loadings were above the recommended value of 0.60 [48]; all Cronbach’s alphas were above 0.70, which is the value recommended in the literature to accept reliability [48]; the composed reliability index was also above 0.70 [47]; and the average variance extracted (AVE) values were superior to the recommended value of 0.50 [47].

The discriminant validity was confirmed using two methods. Both methods are presented in Table 3. This table includes the average variance extracted in the diagonal. Firstly, the confidence interval for the inter-factor correlations was calculated (see Table 3, above the diagonal). Secondly, the variance shared by each pair of factors was also calculated (See Table 3, below the diagonal).

**Table 3:** Discriminant validity.

AVE	SATIS	SELFCON	PSV	IC	VSNBI	VSNCC
SATIS	**0.67**	[0.5–0.66]	[0.07–0.29]	[0.19–0.41]	[0.01–0.21]	[0.14–0.36]
SELFCON	0.32	**0.71**	[0.48–0.66]	[0.02–0.24]	[0.28–0.48]	[0.31–0.52]
PSV	0.03	0.33	**0.53**	[0.19–0.39]	[0.27–0.49]	[0.15–0.36]
IC	0.10	0.02	0.09	**0.78**	[0.06–0.27]	[0.37–0.56]
VSNBI	0.01	0.07	0.14	0.03	**0.63**	[0.15–0.37]
VSNCC	0.06	0.17	0.07	0.22	0.07	**0.63**

The diagonal represents the average variance extracted AVE. Above the diagonal is the 95% confidence interval for the inter-factor correlations. Below the diagonal is the variance shared by each pair of factors (squared correlation). Meaning of the abbreviations in Table 3: SATIS: Satisfaction; SELFCON: Self-congruity; PSV: Perceived social value; IC: Interpersonal Connections on Virtual Social Networks; VSNBI: Use of Virtual Social Networks as a source of information; VSNCC: Use of Virtual Social Networks to create content. Source: own elaboration

As observed in Table 3, the discriminant validity is verified, since the value “1” does not appear in any of the ninety-five percent confidence intervals for correlation estimations between the pairs of factors. Furthermore, the variance shared between each pair of constructs was below the corresponding variance extracted indexes [47].

### 4.3. Structural model

**Fig 2:**
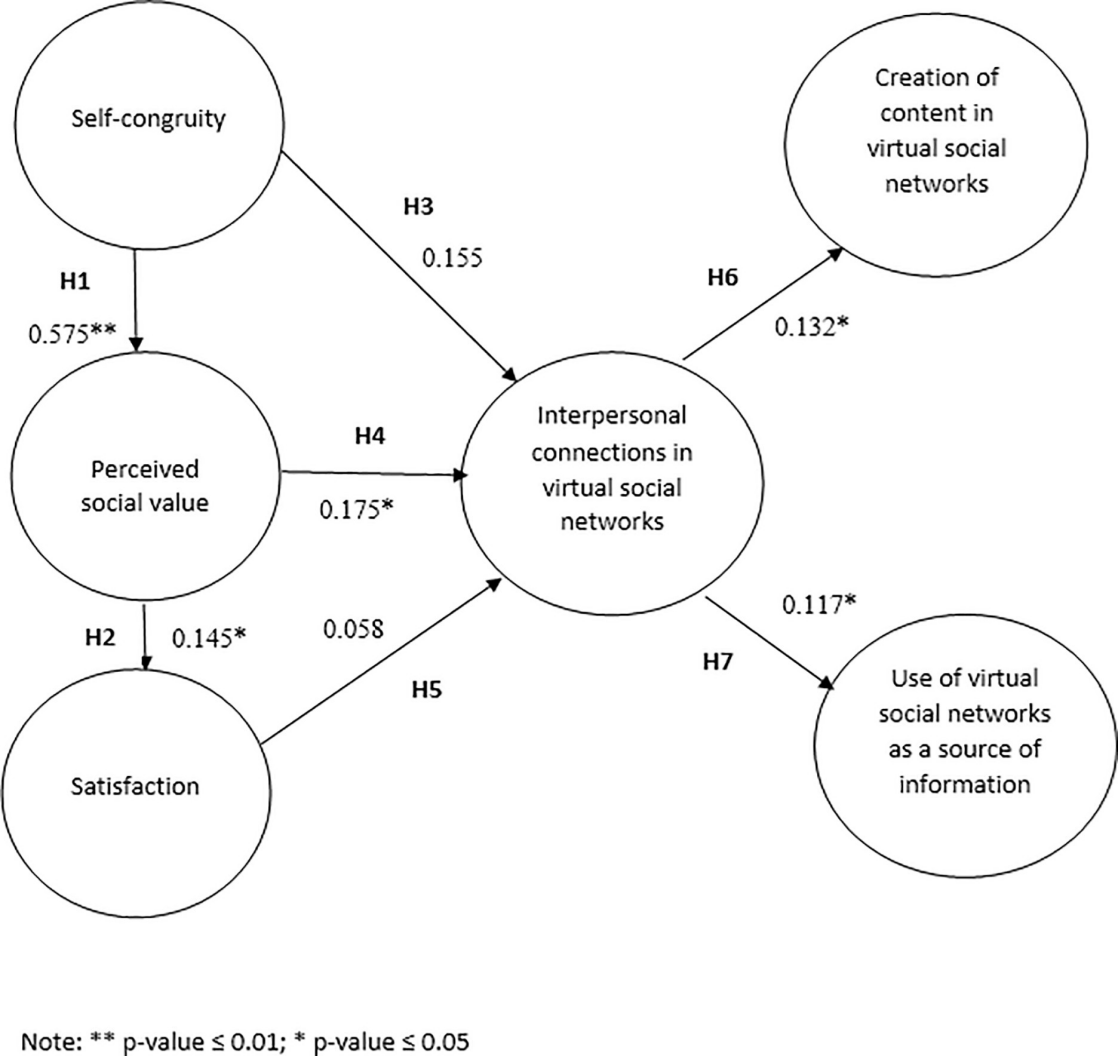
Hypotheses verification. Source: own elaboration.

The hypotheses verification is presented in Fig 2, which shows the relation between the constructs; the standardized coefficients (β); and the T-value for every relation (t); and the Cohen’s *d* to calculate effect size for every relationship in the model (d).

A t-value of 7.639, a structural coefficient (β) of 0.575 and an effect size of 1.264 (Cohen’s d) indicate that self-congruity influences the perceived social value by Generation Y travelers, at 0.01 level of significance (H1 confirmed). A t-value of 2.058, a structural coefficient (β) of 0.145 an effect size of 0.340 (Cohen’s d) indicate that perceived social value influences the satisfaction felt by Generation Y travelers, at 0.05 level of significance (H2 confirmed). A t-value of 2.361, a structural coefficient (β) of 0.175 and an effect size of 0.390 (Cohen’s d) indicate that perceived social value influences the interpersonal connection created by Generation Y travelers in their virtual social networks, at 0.05 level of significance (H4 confirmed).

Furthermore, a t-value of 2.442, a structural coefficient (β) of 0.132 and an effect size of 0.404 (Cohen’s d) indicate that the interpersonal connection in the virtual social networks influence the use of these sites to search for information, at 0.05 level of significance (H6 confirmed). Finally, a t-value of 2.266, a structural coefficient (β) of 0.117 and an effect size of 0.375 (Cohen’s d) indicate that the interpersonal connection in the virtual social networks influence the use of these sites to create content about the trip, at 0.05 level of significance (H7 confirmed).

Regarding the association between self-congruity and satisfaction, a t-value of 1.552, a structural coefficient (β) of 0.145 and an effect size of 0.256 (Cohen’s d) indicate that the relationship between these constructs cannot be accepted in this context of research (H3 not confirmed). It can also be observed that the relationship between the satisfaction and the creation of interpersonal connections on virtual social networks by Generation Y travelers could not be accepted, based on a t-value of 0.140, a structural coefficient (β) of 0.058 and an effect size of 0.023 (Cohen’s d) for this relationship (H5 not confirmed).

## 5. Conclusions

### 5.1. Discussion

This research focused on the relationship between self-congruity, perceived social value and the interpersonal connections in virtual social networks. It also examined the influence of the interpersonal connections on the use of virtual social networks to create content and as a source of information. The study is centered on Generation Y tourists.

First of all, the results indicate that there is a relationship between self-congruity and perceived social value in this context of research. Secondly, the results show that the perceived social value influences the satisfaction felt by Generation Y travelers. Thus, when young tourists believe that the tourism experience is congruent with their self-concept, they perceive a higher social value. Subsequently, these tourists are more satisfied with the experience when they perceive a higher social value.

Ballesteros and Ramírez [8] pointed out that social identities play an important role in the development of heritage tourism. However, the connections made in their study were theoretical, and no empirical research was performed to support the affirmations. Correia et al. [3] used a qualitative study, based on 36 Portuguese citizens. The authors indicate that the social context moderates the ways in which the individuals perceive their trip. Through a different approach, the present research provides similar conclusions. In this case, it is established that self-congruity influences the social value perceived by the travelers during their tourism experience, and the social value influences the tourists’ satisfaction.

Hosany and Martin [30] analyzed the effect of self-congruity on some post-trip consequences. Although the authors did not measure the association between self-congruity and perceived social value, they pointed out that symbolic consumption influences satisfaction. Kim and Jang [4] affirmed that some individuals have preferences for experiences that provide social value. Also, they suggested that the perceived social value can affect some behavioral intentions. The findings of the present research add knowledge in connection with previous studies that focus on consumers’ identity and social value, in this case, in the area of tourism. When it comes to the relationship between these constructs and the creation of interpersonal connections in virtual social networks, it is observed that social value influences this consumer behavior.

The relationship between self-congruity and the interpersonal connections in virtual social networks was not confirmed. This hypothesis was proposed based on the study by Kim et al. [5]. These authors indicated that identity is an important factor that explains seniors’ revisit intention to virtual social networks for tourism-related purposes. Based on the conclusions by Kim et al. [5], and the results of the present research, it could be suggested that the congruence with the tourism experience does not influence the use of virtual social networks. The resemblance with other users, with whom the tourist shares similar interests and identities, seems to be the factor that influences the interpersonal connections in virtual social networks, but not the congruence between the tourists’ self-concept and the experience.

Based on the results, it could not be accepted that the tourists’ satisfaction influences the interpersonal connections in virtual social networks. This hypothesis was proposed based on previous studies [39]. These authors suggested that young consumers might talk and share more information with other people when they feel satisfied with a specific part of their trip. Although these authors did not measure the relationship through an empirical research, we planned the hypothesis based on their affirmations. In the present research, the participants were asked about their overall satisfaction with their trips, and this construct seems not to influence the connections made on their virtual social networks. Consumers create content about specific aspects of the trip [5]. Perhaps, the satisfaction with particular attractions or places during their vacations influences the use of virtual social networks, but not their overall satisfaction with the tourism experience.

Furthermore, Nusair et al. [1] indicated that, when young tourists perceive that a trip provided value, they use their virtual social networks to talk about topics of interest with geographically dispersed consumers with whom they shared the experience. These authors analyzed a series of constructs to examine some antecedents of the social interaction on the internet by Generation Y travelers. Specifically, they examined how innovativeness, information sharing, perceived risk and perceived utility are antecedents of social interaction, trust, and loyalty. In relation to the present research, it can be added that perceived social value is an antecedent of the social interaction in virtual social networks, in this case, based on the fact that Generation Y travelers create interpersonal connections when they travel, which influence a more frequent use of these tools.

These results connect with other previous findings. For instance, Hamilton et al. [33] established a relationship between the interaction in virtual social networks, perceived value and satisfaction. In connection with these previous findings, the results of the present research indicate that the social dimension of the perceived value influences the interpersonal connections created in virtual social networks. Furthermore, when the consumers interact with some users with whom they shared the experience, they will probably ask them some advices and share pictures, videos and other information.

Xiang and Gretzel [43] measured the influence of interpersonal connections among users on the use of virtual sites as sources of information. The authors used a data mining exercise and did not focus on a specific generation. Hence, the present research adds new findings regarding the influence of interpersonal connection on the use of virtual social networks.

### 5.2. Managerial implications

This paper offers some guidelines for marketers regarding the interpersonal connections made by Generation Y travelers in their virtual social networks. Thus, tourism companies and public institutions should take into account that, when an experience is congruent with the tourists’ self-concepts, they will perceive a higher social value, which is a variable that positively affects the interpersonal connections in their virtual social networks.

Nowadays, the consumers have gained some power over the companies on the internet. They create a lot of content about brands, services and destinations, and the users’ advices are perceived as reliable by other consumers [12]. The interpersonal connections in the virtual social networks influence a higher use of these sites, which leads to content creation. The content could be of interest for other tourists, who might change their perception of a particular destination or a tourism experience.

Hence, tourism companies should investigate to understand Generation Y travelers’ motivations, interests and values. Subsequently, the companies should check the similarities regarding the interests and values among their young clients, and persuade relationships during the trip. The tourists with similar identities can be located together during lunch and dinner, and participate in activities specially provided for them. The company should motivate them to visit specifics museums or excursions at the same time, where these tourists may bond based on their shared interests and identities.

The efforts by the companies to form these connections can lead to positive commentaries on the virtual social networks. Consequently, the tourists will show pictures and videos of the destination when they recreate the experience together on the internet. This information can have a more powerful impact on other consumers than the messages posted by the companies in the virtual social networks.

### 5.3. Limitations and future lines of research

This study presents some limitations. First of all, the research is centered on highly educated consumers. It is not known if the results would be similar in a study centered on travelers who do not study at a university level. It would be interesting to examine the approaches of the present research considering different levels of education.

The study did not include any other control for confounding among the respondents of the research. For instance, it could be possible that wealthier students travel to fancier destinations, and that this fact influences a higher perceived social value and the presentation of the information in their virtual social networks. This analysis could provide further conclusions that were not measured in the present research. Therefore, it would be interesting to include a control for confounding in future studies that examine the relationships analyzed in this research.

Furthermore, the sample is based on Spanish travelers. It would be interesting to examine the connections of these constructs in a research focused on tourists from different countries. In addition, the study did not discriminate among different kind of trips (adventure, cultural, etc.). Hence, the results of this research could be compared with future studies that focus on specific trips.

## Supporting information

S1 File Data set. (XLSX)
